# Insights into Candidate Pathways Associated with Multi-Pistil Formation in Chinese Chestnut (*Castanea mollissima* Bl.): Evidence from Anatomy, Physiology, and Molecular Biology

**DOI:** 10.3390/biology15171459

**Published:** 2026-08-26

**Authors:** Jiaxuan Xie, Sujuan Guo, Ruijie Zheng

**Affiliations:** 1 National Key Laboratory of Efficient Production of Forest Resources, Beijing Forestry University, Beijing 100083, China; jiaxuanxiebjfu@sohu.com; 2 Key Laboratory of Forest Cultivation and Protection, Ministry of Education, Beijing Forestry University, Beijing 100083, China; 3 Liaoning Economic Forest Research Institute, Liaoning Academy of Agricultural Sciences, Shenyang 116031, China; zhengruijie2006@sohu.com

**Keywords:** *Castanea mollissima* Bl., multi-pistil phenotype, floral development, phytohormones, transcriptome, metabolome

## Abstract

The number of pistils determines the yield of Chinese chestnut (*Castanea mollissima* Bl.). However, the candidate pathways associated with formation of multi-pistil female flower clusters (MFF) remain largely unclear. This study integrated morphological, anatomical, transcriptomic, and targeted phytohormone metabolomic approaches to compare MFF and normal female flower clusters (NFF). The results showed that MFF exhibited longer basal bracts with fewer basal trichomes. The critical stage for multi-pistil phenotype formation occurred during pistil primordium differentiation, when additional floral meristems continuously expanded and differentiated into new flower primordia. Auxin (IAA), cytokinin (CTK), jasmonic acid (JA), and gibberellin (GA) were identified as potentially associated with this. Candidate genes associated with hormone biosynthesis, signal transduction, and core transcription factors regulating floral organ development at this key developmental stage were also identified. The findings of this study provide insights into the potential regulatory mechanisms underlying the differentiation and development of MFF.

## Simple Summary

Chinese chestnut is an economically important nut crop, and its yield is strongly influenced by the pistil number of each female inflorescence. However, the molecular basis for the formation of multi-pistil female flower clusters remains poorly understood. In this work, comparative analyses between multi-pistil and normal female flower clusters were performed by combining anatomical observation, targeted phytohormone metabolomics, and transcriptome sequencing. The observations indicate that pistil primordium differentiation is the critical stage for multi-pistil formation, during which extra floral meristems expand continuously and give rise to new flower primordia. Changes in several plant hormones, including auxin, cytokinin, jasmonic acid, and gibberellin, were observed alongside this process. We also identified a number of candidate genes that may be involved in hormone regulation and flower development. These findings provide a useful foundation for further research and may support future breeding efforts to improve chestnut yield.

## 1. Introduction

Floral organ development in plants is coordinately regulated by genetic and environmental factors [1], with the number of floral organs serving as a primary determinant of crop yield [2]. The multi-pistil trait has been documented in various species, including Japanese apricot (*Prunus mume* Sieb. et Zucc.) [3], sweet cherry (*Prunus avium* L.) [4], rapeseed (*Brassica napus* L.) [5], wheat (*Triticum aestivum* L.) [6,7,8], rice (*Oryza sativa* L.) [9], rye (*Secale cereale* L.) [10], and tomato (*Solanum lycopersicum* Mill.) [11], where it often significantly affects their yield. This phenomenon is typically associated with floral meristem (FM) abnormalities. The timely termination of the FM is a prerequisite for normal floral organ development [12]; consequently, any disruptions to this process can lead to the formation of ectopic pistils [13]. Environmental signals [4], genetic mutations [6], transcriptional regulation [14], and endogenous hormone levels [3,8] cooperatively regulate FM termination.

Plant hormones are critical regulators of FM activity and floral organ patterning [15]. For instance, in multi-pistil Japanese apricot, zeatin (ZT) and IAA levels are elevated during early pistil differentiation, whereas GA_3_ levels are reduced [3]. Conversely, multi-pistil inflorescences in coconut (*Cocos nucifera* L.) show reduced levels of IAA, zeatin riboside (ZR), and GA, while abscisic acid (ABA) and JA are elevated [16]. Furthermore, exogenous application of cytokinin analogs (e.g., BA) or GA_3_ can increase the incidence of double pistils in sweet cherry [14]. Similarly, high nitrogen availability has been shown to increase tomato carpel number by elevating CTK and GA levels while reducing IAA content [17]. JA has also been implicated in regulating floral organ development in rice [18], persimmon (*Diospyros kaki* Thunb.) [19], and Chinese chestnut [20]. Collectively, these studies indicate that phytohormones are critical regulators of floral organ number and exhibit species-specific effects. These hormonal effects are often mediated by the differential gene expression involved in hormone biosynthesis and signaling pathways [21].

Transcription factors (TFs) are equally pivotal in maintaining FM activity and organ patterning [22]. *WUSCHEL* (*WUS*) is required for stem cell maintenance in the meristem [23]. Studies on wheat tri-pistil mutants [6] and multi-pistil *P. mume* [24] have demonstrated that *WUS* overexpression leads to the formation of ectopic pistil primordia [6]. In *Arabidopsis thaliana* (L.) Heynh, the regulatory framework shows that the floral organ identity gene *AGAMOUS* (*AG*) activates the downstream repressor *KNUCKLES* (*KNU*) to terminate *WUS* expression, thereby ensuring FM arrest [25]. In contrast, *APETALA2* (*AP2*) functions antagonistically to *AG* to maintain stem cell homeostasis [26]. Furthermore, KNOX-class TFs, which are repressed by upstream MADS-box genes, play a vital role in regulating pistil number [14], while the NAC and TCP families contribute to the regulation of organ boundary and cell proliferation.

Chinese chestnut is an economically important nut-producing tree. Typically, a female chestnut flower cluster contains one to three pistils [27], and low female flower numbers and an unbalanced sex ratio often limit overall yield [28]. In our previous germplasm survey, we found a unique Chinese chestnut line with a multi-pistil trait, in which specific flower clusters produce more than three pistils. Most female flower clusters on the upper canopy bear more than three female flowers, whereas flower clusters in the middle and lower canopy of the same tree develop into normal single-pistil inflorescences, and this trait holds potential for yield improvement. Despite its importance, the mechanisms underlying multi-pistil formation in Chinese chestnut remain largely unclear.

Although previous studies have investigated the anatomical characteristics of female flower differentiation in Chinese chestnut, their focus has been primarily on the dynamic changes during different floral organ differentiation stages [27], with no report specifically addressing the anatomical features underlying multi-pistil formation. Furthermore, previous molecular studies have largely focused on the functional characterization of specific gene families involved in floral organ development, such as the MADS-box genes regulating floral organ identity [29,30], the GA-mediated *SPL* gene family and miR156 affecting floral development [31], and the JA-mediated *JAZ*/*MYC2* signaling pathway involved in female flower bud differentiation [32]. However, potential molecular and hormonal pathways associated with multi-pistil formation remain unexplored. Therefore, to characterize cytological features and identify physiological and molecular pathways potentially associated with multi-pistil differentiation in Chinese chestnut, integrated analyses were performed using morphological, anatomical, targeted phytohormone metabolomic, and transcriptomic datasets. The outputs of this work provide a scientific reference and a theoretical basis for generating hypotheses about the potential regulatory network of multi-pistil development in this species.

## 2. Materials and Methods

### 2.1. Plant Materials

The experiment was conducted during 2024–2025 at the Chestnut Breeding and Cultivation Base of Beijing Forestry University (Qianxi County, Hebei Province, China; 118°54′ E, 40°18′ N) [33]. This study used the multi-pistil chestnut germplasm ‘WQ’, which was grafted onto 3-year-old seedling rootstocks in 2013. Trees were spaced at 4 m × 4 m and managed under standard orchard conditions. Ten healthy and uniform trees were selected and tagged for this study.

### 2.2. Morphological Observation

From late April to early June in both 2024 and 2025, 50 vigorous bearing shoots were collected from the upper outer canopy of the tagged trees. All mixed inflorescences on these shoots were tagged and numbered. Starting from the emergence of the mixed inflorescences, the external morphology of the inflorescences was observed and photo-graphed every 3–5 days until the female flowers were fully open. After full bloom, based on the number of female florets per female flower cluster, the inflorescences were classified into multi-pistillate type (>3 florets per cluster) and normal type (≤3 florets per cluster). The female flower clusters on these inflorescences were designated as MFF and NFF, respectively. The morphological differences between MFF and NFF at different developmental stages were compared during flower development.

### 2.3. Anatomical Observation

From late April to early June 2025, based on the morphological differences described in Section 2.2, mixed inflorescences of multi-pistillate and normal types were collected from the upper part of the outer canopy every 3–5 days. The female flower clusters at the base of the inflorescences were dissected under a stereomicroscope and designated as MFF and NFF. Samples were fixed in FAA solution (Biosharp, Hefei, China) for 24 h and dehydrated through a graded ethanol series. For SEM, samples were critical-point-dried, sputter-coated, and observed using a JSM-6700F SEM (JEOL, Akishima, Japan). For light microscopy, samples were cleared in xylene, embedded in paraffin (melting point 58–60 °C, Sinopharm Chemical Reagent Co., Ltd., Shanghai, China), sectioned at a thickness of 8 μm (Leica RM2265, Leica Microsystems GmbH, Wetzlar, Germany), and stained with safranin and fast green (Solarbio, Beijing, China) before observation under an optical microscope (Leica DMi8, Leica Microsystems GmbH, Wetzlar, Germany).

### 2.4. Transcriptome Sequencing and Analysis

Samples were collected simultaneously with those in Section 2.3. Based on the observations from Section 2.2 and Section 2.3, mixed inflorescences of multi-pistillate and normal types at the pistil primordium differentiation stage were selected. The female flower clusters at the base of the inflorescences were dissected, designated as MFF and NFF, immediately frozen in liquid nitrogen, and stored at −80 °C. For each group (MFF and NFF), three biological replicates were prepared, with each replicate consisting of pooled female flower clusters from multiple trees. Total RNA was extracted with TRIzol (Invitrogen, Carlsbad, CA, USA), and library construction and sequencing were performed on the Illumina NovaSeq X Plus platform (Illumina, Inc., San Diego, CA, USA). Raw data were aligned to the Chinese chestnut reference genome (https://www.ncbi.nlm.nih.gov/datasets/genome/GCA_000763605.2/, accessed on 25 August 2026). Transcript abundance was measured in TPM, and differentially expressed genes (DEGs) were screened with DESeq2 (v1.42.0; Bioconductor Project, Seattle, WA, USA), with thresholds of log_2_FC > 1 and adjusted *p* < 0.05. Functional enrichment analyses (GO and KEGG) were performed using GOATOOLS (v1.5.1; National Center for Biotechnology Information, Bethesda, MD, USA) and custom R scripts (v4.2.2; R Foundation for Statistical Computing, Vienna, Austria) (*p* < 0.05).

### 2.5. Targeted Hormonal Metabolomics Analysis

The samples were the same as those in Section 2.4. For each group (MFF and NFF), three biological replicates were prepared, with each replicate consisting of pooled female flower clusters from multiple trees. A 100 mg aliquot of each sample was ground and extracted with 80% methanol containing an SA-D4 internal standard. Detection was performed using a UHPLC-Qtrap system (QTRAP 7500, SCIEX LLC, Framingham, MA, USA). Chromatographic separation was achieved on a Waters HSS T3 column (2.1 × 100 mm, 1.7 μm, Waters Corporation, Milford, MA, USA). The mobile phases comprised 0.05% formic acid in water (A) and 0.05% formic acid in methanol (B). Ion fragments were identified using the Sciex OS software (v3.0, SCIEX LLC, Framingham, MA, USA), and concentrations were calculated against standard curves. Metabolites with |fold change| ≥ 1 and *p* < 0.05 were considered differentially accumulated.

### 2.6. qRT-PCR Validation

To validate the reliability of the transcriptomic data, 14 DEGs were selected for qRT-PCR, covering the IAA (*YUCCA4*, *PIN5*, *GH3.17*), CTK (*CKX1*, *ARR15*), JA (*LOX21*, *JAZ*), and GA (*GA20ox*, *GID2*) pathways, as well as transcription factors involved in meristem activity and floral development (*WUS*, *HSBH1*, *YAB1*, *TCP15*, and *NAC104*). Total RNA was extracted using TRIzol reagent. cDNA was synthesized from 1–2 μg of total RNA using All-in-One RT MasterMix (with dsDNase) (Heyuan Liji, Heyuan, China). qRT-PCR was performed on a Bio-Rad CFX Connect system (Bio-Rad Laboratories, Hercules, CA, USA) using 2× qPCR Mix (SYBR Green; Heyuan Liji, Heyuan, Chin) with gene-specific primers synthesized by Sangon Biotech (Shanghai, China) and purified by PAGE. Relative expression levels were calculated using the 2^−ΔΔCt^ method [34], with Chinese chestnut actin serving as the internal reference gene [35]. Primer sequences are provided in Table S1.

### 2.7. Statistical Analysis

Data were organized using the Excel software. Student’s *t*-test was performed in SPSS 24.0 to compare groups, and *p* < 0.05 was considered statistically significant. Data visualization was conducted using Origin 2019.

## 3. Results

### 3.1. Morphological and Anatomical Analysis of Multi-Pistil Formation in Chinese Chestnut

#### 3.1.1. Comparative Morphological Observation of MFF and NFF

**Figure 1:**
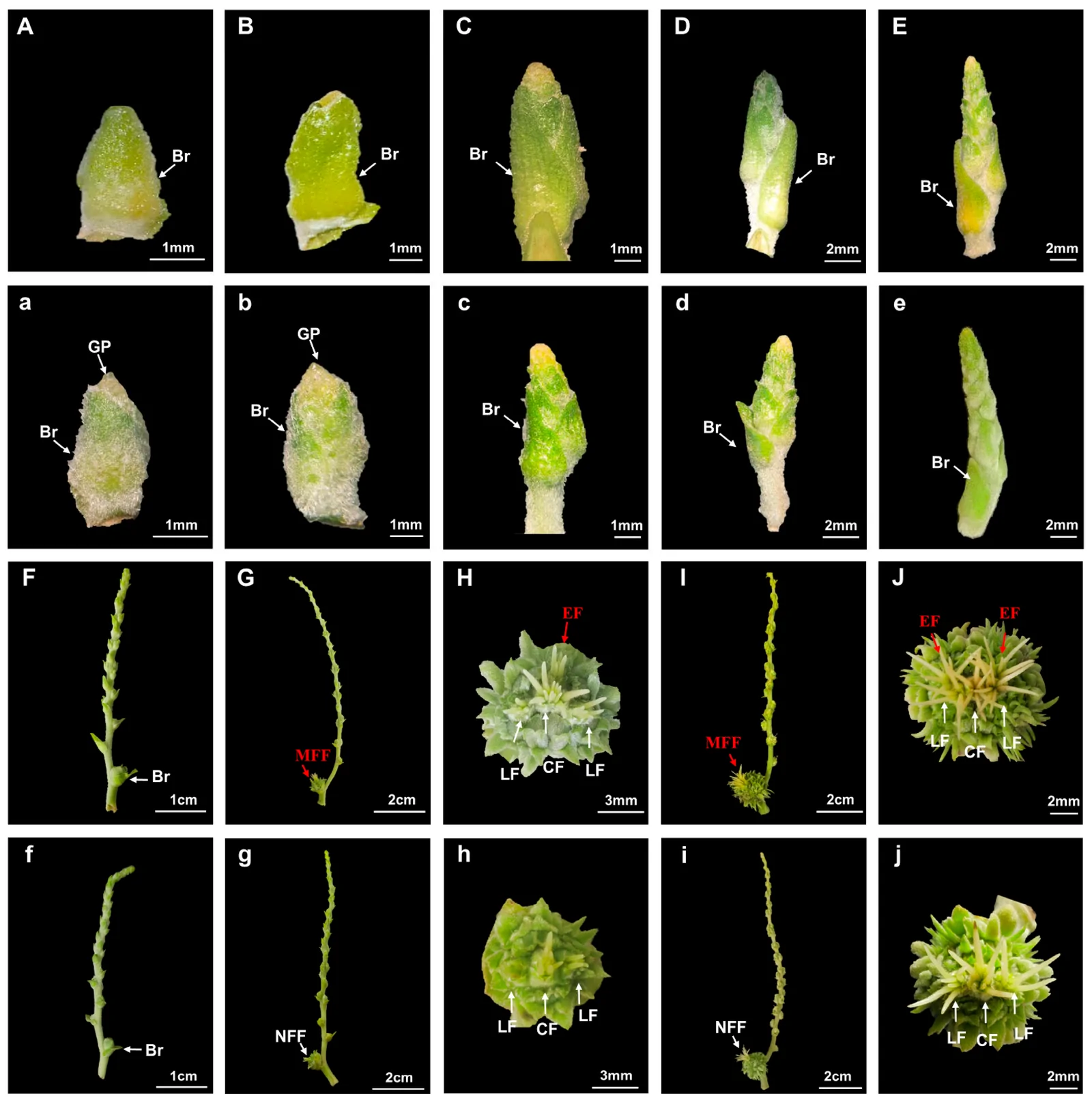
External morphological observation on the development process of two types of female flower clusters in ‘WQ’. (**A**,**a**) Bract differentiation Stage; (**B**,**b**) bract enlargement stage; (**C**,**c**) bract elongation stage; (**D**,**d**) inflorescence elongation stage; (**E**,**e**) female flower formation stage; (**F**,**f**) female flower swelling stage; (**G**,**H**,**g**,**h**) multiple female flower emergence stage; (**I**,**J**,**i**,**j**) stigma bifurcation stage; red arrows indicate multi-pistil female flower clusters and extra florets. Br, bract; GP, growing point; MFF, multi-pistil female flower clusters; NFF, normal female flower clusters; CF, central floret; LF, lateral floret; EF, extra floret.

Continuous observation of external morphology showed that the differentiation of ‘WQ’ female flower clusters began in late April and lasted approximately 40 days, which could be divided into eight developmental stages (Figure 1; Table 1).

- During the bract differentiation and bract enlargement stages, mixed inflorescences emerged with swollen basal portions covered by bracts. The basal bract length of MFF was significantly greater than that of NFF (Table 1). Specifically, the inflorescences of MFF were entirely wrapped by sparsely hairy bracts, whereas the shoot apical meristems of NFF were exposed and covered with dense hairs (Figure 1A,B,a,b), with no significant difference in inflorescence length between the two types.
- From the bract elongation stage to the female flower formation stage, both bracts and inflorescences underwent rapid elongation growth. The basal bract length of MFF was significantly longer than that of NFF (*p* < 0.05) during this period. The bracts of MFF enveloped most of the inflorescence structure, while those of NFF only covered the basal part of the inflorescence (Figure 1C–E,c–e; Table 1).
- At the female flower enlargement stage, the basal bracts of both MFF and NFF ceased growth and curled outward, and MFF still exhibited extremely significantly longer basal bracts than NFF (Figure 1F,f; Table 1).
- From the multiple female flower emergence stage to the stigma bifurcation stage, the outer bracts of female flower clusters abscised, and the inflorescence length reached its maximum value. At this stage, only three florets with stigmas protruding from the cupule were observed in NFF, while more than three protruding florets were detected in MFF (Figure 1G–J,g–j).

#### 3.1.2. Microscopic and Anatomical Differences Between MFF and NFF

The correspondence between internal anatomical structures and external morphology was determined by scanning electron microscopy (SEM) and paraffin section observations, allowing for the division of MFF differentiation into eight developmental stages (Table 1). Observations showed that within each female flower cluster, the central flower differentiated first, followed by the lateral flowers, with the additional florets initiating last.

**Figure 2:**
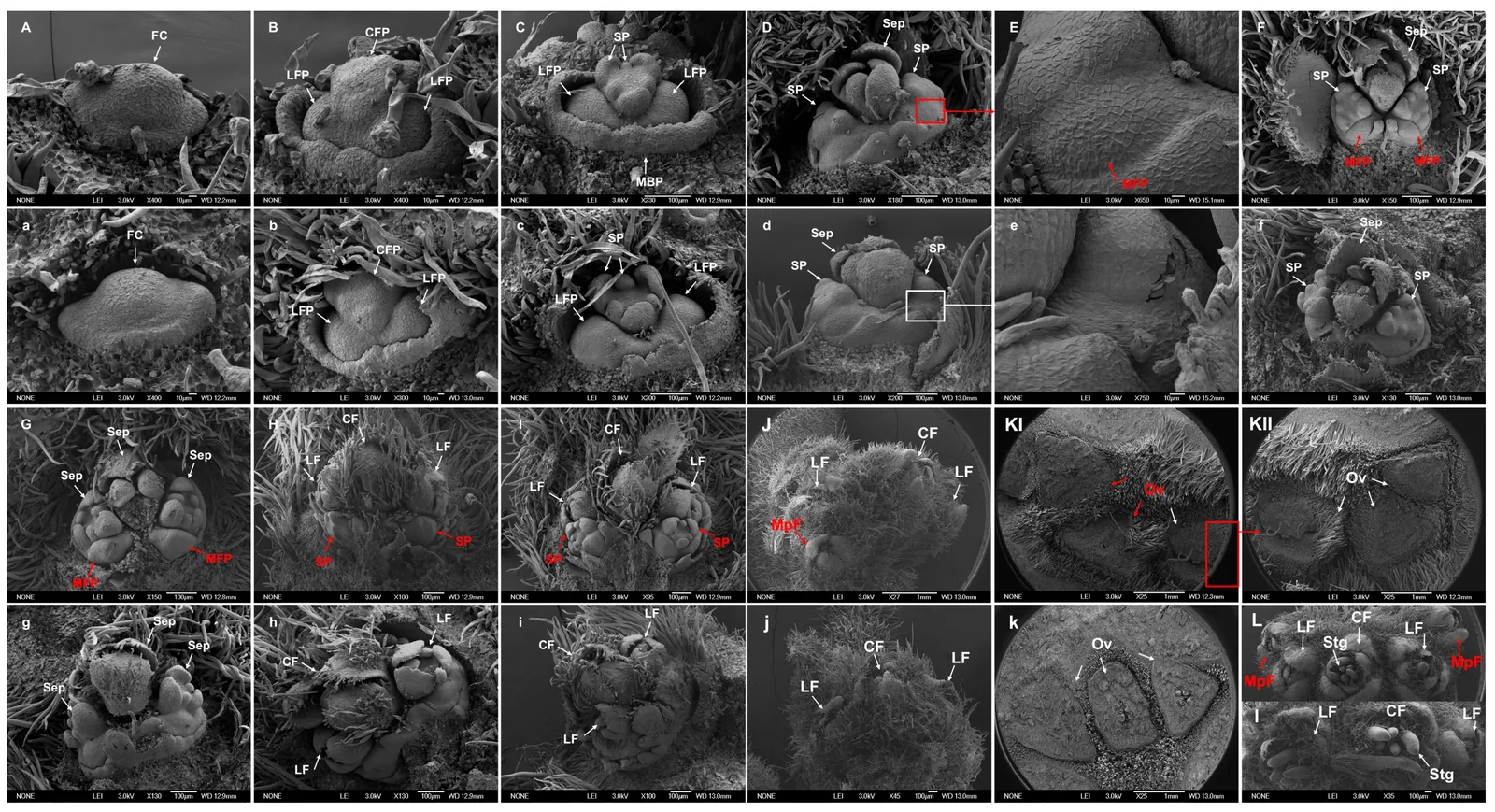
Scanning electron microscopic observation of floral cluster development of two types in ‘WQ’. (**A**–**L**) Microstructure of each developmental stage of MFF; (**a**–**l**) microstructure of the corresponding developmental stage of NFF. (**A**,**a**) Floral cluster primordium differentiation stage; (**B**,**b**) flower primordium differentiation stage; (**C**,**c**) sepal primordium differentiation stage; (**D**,**E**,**d**,**e**) stamen primordium differentiation stage; (**F**,**G**,**f**,**g**) pistil primordium differentiation stage; (**H**–**J**,**h**–**j**) stigma primordium differentiation stage; (**KI**,**KII**,**k**) ovary formation stage, (**KI**), left ovary structure of MFF floral cluster; (**KII**), right ovary of the same cluster in the same field of view; red box indicates the partial structural region shown in subpanel KI; (**L**,**l**) flowering stage. FC, floral cluster primordium; CFP, central flower primordium; LFP, lateral flower primordium; MBP, middle bract primordium; SP, sepal primordium; Sep, sepal; MFP, additional flower primordium of multi-pistil flower; CF, central flower; LF, lateral flower; Ov, ovary; MpF, multi-pistil flower; Stg, stigma; boxes with arrows indicate magnified views; red arrows mark the extra primordia, multi-pistil flowers, and differentiated ovary structures specific to MFF, highlighting key morphological distinctions between MFF and NFF.

When the external morphology was at the bract primordium differentiation stage through the bract elongation stage, the female flower cluster primordium differentiated within the basal bracts of the inflorescence (Figure 2A,a). Subsequently, flower primordia of the central and lateral flowers formed successively on the female flower cluster primordium (Figure 2B,b), with the central flower differentiating sepal primordia first (Figure 2C,c). At these stages, no obvious structural differences were observed between MFF and NFF.

**Figure 3:**
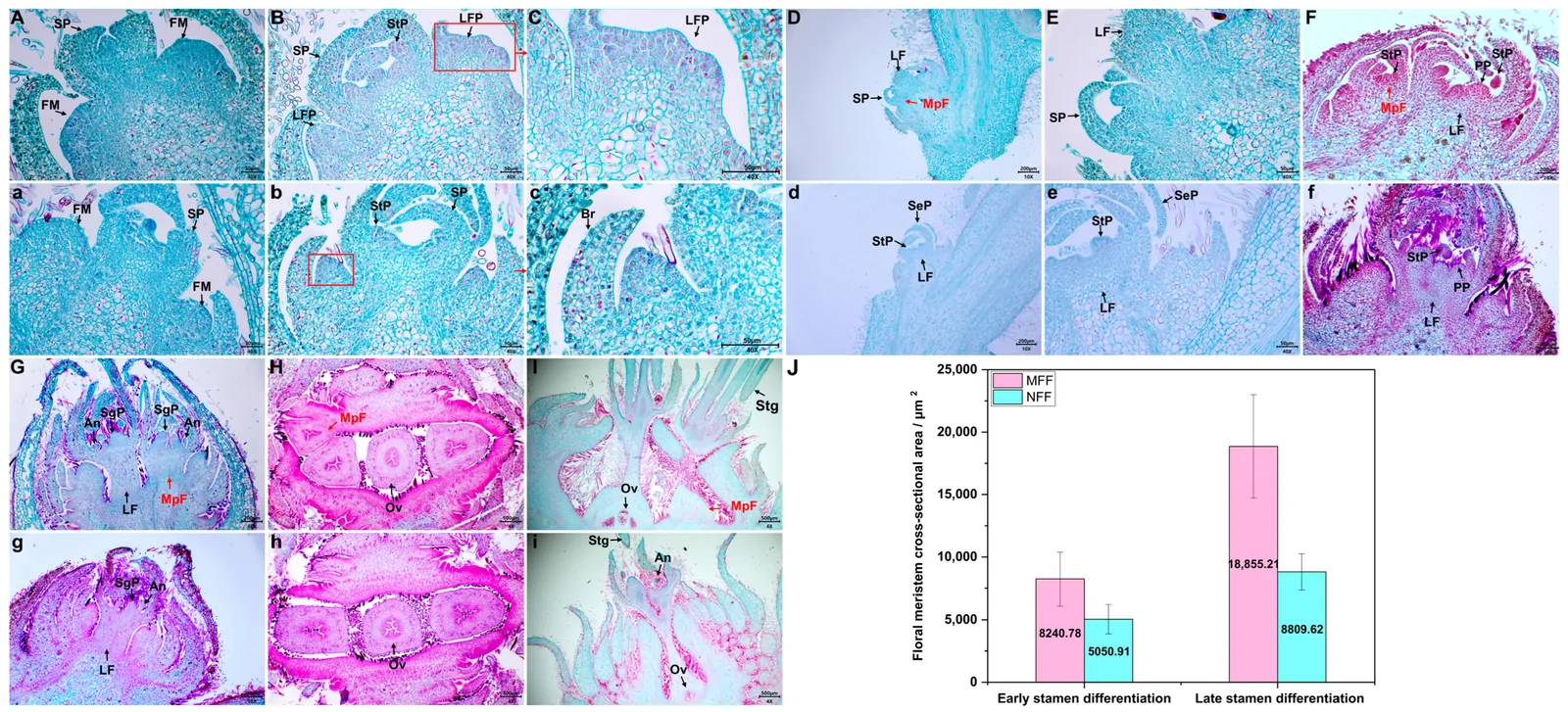
Anatomical structure (paraffin section) and floral meristem cross-sectional area analysis of two types of floral clusters in ‘WQ’. (**A**–**I**) Paraffin section anatomical structures of MFF at each developmental stage; (**a**–**i**) paraffin section anatomical structures of NFF at each developmental stage. (**A**,**a**) Stamen primordium differentiation stage; (**B**,**C**,**b**,**c**) pistil primordium differentiation stage; red box indicates the magnified region of subpanels B and b; (**D**–**G**,**d**–**g**) stigma primordium differentiation stage; (**H**,**h**) ovary formation stage; (**I**,**i**) flowering stage; (**J**) comparison of floral meristem cross-sectional area between MFF and NFF. FM, floral meristem; Br, bract; StP, stamen primordium; PP, pistil primordium; SgP, stigma primordium; An, anther; red arrows indicate multi-pistil flower, marking the characteristic structure unique to MFF.

When the external morphology reached the inflorescence elongation stage, the central flower differentiated stamen primordia, while the lateral flowers formed sepal primordia. Additional floral meristems (FMs) accumulated on both sides of the lateral flowers in both MFF and NFF (Figure 2D,E,d,e). However, the accumulated FMs in MFF were larger in volume, exhibited darker tissue staining, and showed stronger cell division activity compared to those in NFF (Figure 3A,a,J).

When the external morphology entered the female flower formation stage, the central flower differentiated pistil primordia, while the lateral flowers formed stamen primordia. In MFF, the additional FMs continued to enlarge and differentiated into new flower primordia (Figure 2F,G), with densely packed meristematic cells displaying uniform morphology (Figure 3B,C,J). In contrast, the additional FMs in NFF showed no further differentiation (Figure 2f,g) and contained fewer meristematic cells (Figure 3b,c,J). This stage was identified as the critical period for multi-pistil formation.

When the external morphology entered the female flower enlargement stage to the stigma bifurcation stage, the central and lateral flowers successively formed stigma primordia and ovaries. In MFF, the new flower primordia derived from additional FMs sequentially differentiated into all floral organ whorls and developed into extra florets (Figure 2H–L and Figure 3D–I), with relatively strong cell division activity. In NFF, no extra florets were formed from the additional FMs (Figure 2h–l and Figure 3d–i). Consequently, MFF developed multi-ovary and multi-flower structures, whereas NFF produced only three florets (Figure 2J–L,j–l and Figure 3H,I,h,i).

### 3.2. Transcriptome Analysis

#### 3.2.1. Transcriptome Assessment

Based on the anatomical observations, the pistil primordium differentiation was identified as the critical period distinguishing MFF and NFF. Therefore, female flower clusters of both types at this stage were selected for transcriptome analysis. Transcriptome sequencing of these samples generated 248,274,534 raw reads, with high-quality clean reads accounting for >99% and a reference genome mapping rate of >94% (Table S2). Principal component analysis (PCA) showed good reproducibility within groups and clear separation between groups (Figure S1a). Pearson’s correlation coefficient (PCC) analysis further verified biological replicate reproducibility (Figure S1c). Within-group PCC values were 0.996–0.998 for MFF and 0.983–0.993 for NFF, demonstrating high repeatability. In contrast, low inter-group coefficients (0.449–0.495) showed obvious transcriptomic divergence between MFF and NFF. A total of 2647 DEGs were identified in the MFF vs NFF comparison, including 1504 upregulated and 1143 downregulated (Figure S1b).

#### 3.2.2. GO and KEGG Enrichment Analysis of DEGs

**Figure 4:**
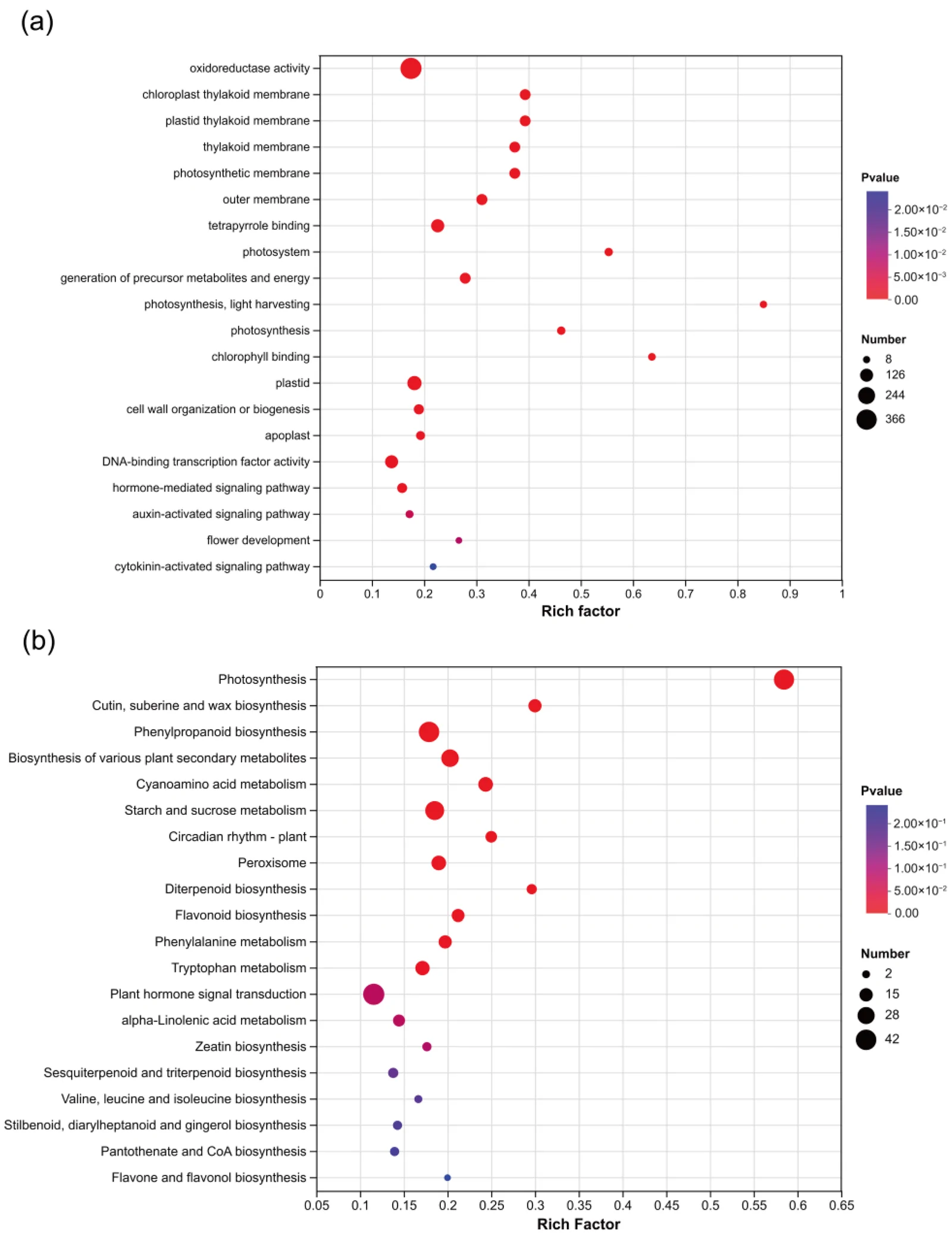
GO and KEGG enrichment analysis of differentially expressed genes. (**a**) GO enrichment analysis; (**b**) KEGG enrichment analysis.

GO and KEGG pathway enrichment analyses were performed on the DEGs between MFF and NFF. GO enrichment results (Figure 4a) showed that the DEGs were mainly enriched in biological processes such as “oxidoreductase activity”, “chloroplast thylakoid membrane”, “cell wall organization or biogenesis”, “DNA-binding transcription factor activity”, “hormone-mediated signaling pathways”, “auxin-activated signaling pathways”, and “flower development”. KEGG enrichment results (Figure 4b) further showed that the DEGs were significantly enriched in “photosynthesis”, “cutin, suberine and wax biosynthesis”, “phenylpropanoid biosynthesis”, “starch and sucrose metabolism”, “diterpenoid biosynthesis”, and “tryptophan metabolism”. In addition, hormone-related pathways such as “plant hormone signal transduction”, “alpha-linolenic acid metabolism”, and “zeatin biosynthesis” were also substantially enriched. These results suggested that differential transcription of carbon metabolism, energy metabolism, secondary metabolism, and hormone signals may play a key regulatory role in multi-pistil formation.

### 3.3. Hormonal Metabolomics Analysis

#### 3.3.1. Analysis of Differential Hormones

KEGG enrichment indicated that many DEGs were associated with phytohormone-biosynthesis-related pathways, including “diterpenoid biosynthesis”, “tryptophan metabolism”, “plant hormone signal transduction”, “alpha-linolenic acid metabolism”, and “zeatin biosynthesis” (Figure 4). Collectively, phytohormones appeared to be closely associated with multi-pistil formation.

**Figure 5:**
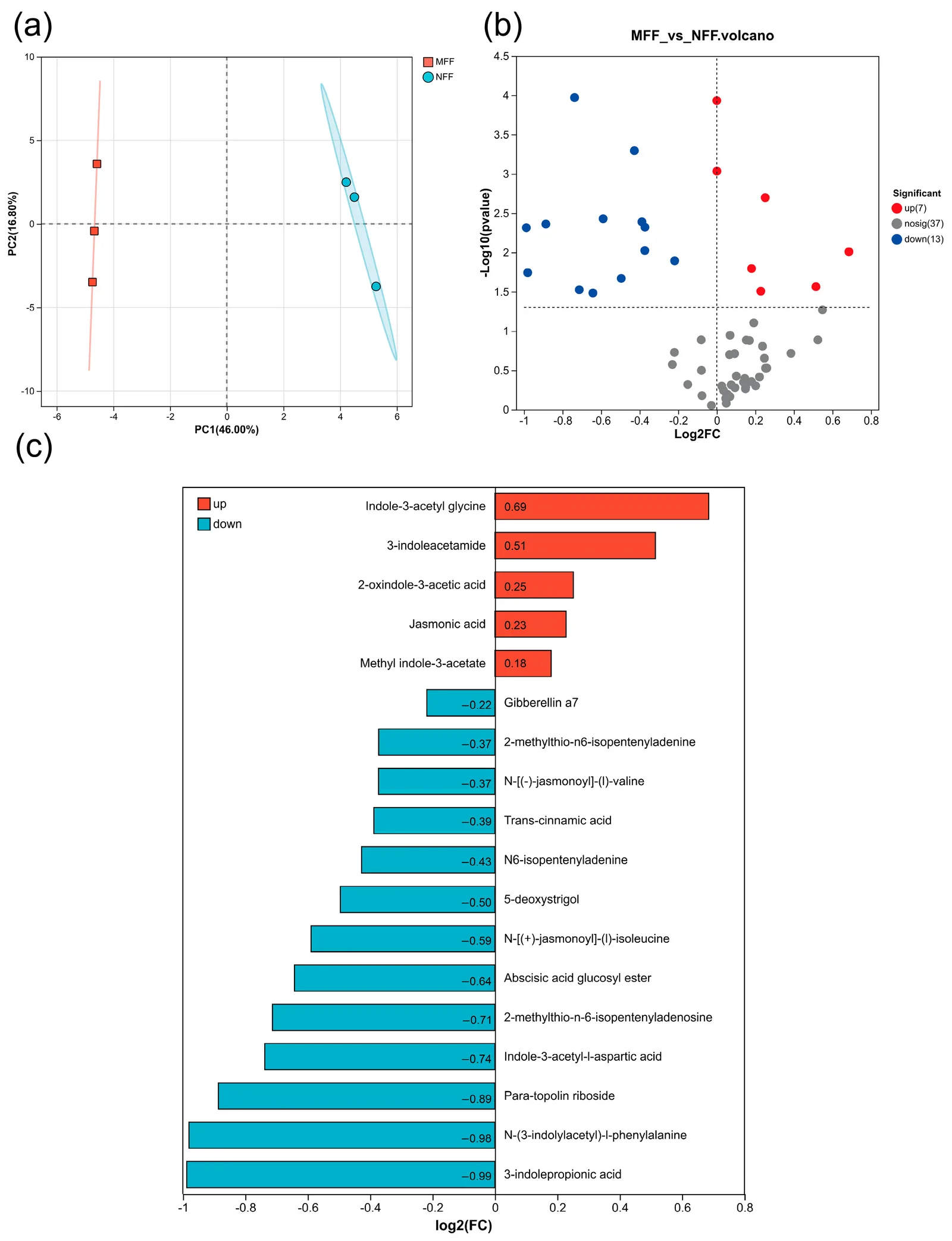
Hormone metabolome analysis between MFF and NFF in Chinese chestnut. (**a**) Principal component analysis (PCA) of hormone samples; (**b**) summary of up- and down-accumulated differential hormones; the y-axis represents −log_10_ (*p*-value), the log-transformed statistical test value for differences in metabolite abundance; higher values correspond to greater statistical significance. Each dot represents one metabolite. Red dots denote significantly up-accumulated metabolites, blue dots denote significantly down-accumulated metabolites, and grey dots represent metabolites with no significant difference. The vertical dashed line denotes log_2_(FC) = 0, and the horizontal dashed line represents the significance threshold of *p*-value = 0.05. (**c**) fold change distribution of differential hormones.

To elucidate the differences in endogenous hormones between MFF and NFF, targeted hormonal metabolomics analysis was performed. PCA clearly separated the two groups (Figure 5a), confirming data reliability. Twenty differentially accumulated metabolites (DAMs) were detected in the MFF vs. NFF comparison, including 7 upregulated and 13 downregulated metabolites in MFF (Figure 5b).

#### 3.3.2. Differential Accumulation of Phytohormones Between MFF and NFF

To further investigate the differential accumulation of phytohormones between MFF and NFF, we performed a quantitative analysis of the fold changes in DAMs (Figure 5c, Table S3).

For auxin, the IAA precursor 3-indolepropionic acid (IPA, log_2_FC = −0.99) and the IAA conjugate N-(3-indoleacetyl)-L-phenylalanine (IAA-Phe, log_2_FC = −0.98) were significantly downregulated in MFF, whereas the glycosylated product indole-3-acetyl glycine (IAA-Gly, log_2_FC = 1.95) was significantly upregulated. Notably, no significant difference was detected in the abundance of active IAA between MFF and NFF. These results suggested that IAA biosynthesis was suppressed while its inactivation pathway was enhanced in MFF.

For cytokinins, the active cytokinin N^6^-isopentenyladenine (iP, log_2_FC = −0.43) exhibited decreased accumulation in MFF, indicating reduced bioactive CTK levels.

For jasmonates, free jasmonic acid (JA, log_2_FC = 0.23) was upregulated, whereas its bioactive derivatives jasmonoyl-isoleucine (JA-Ile, log_2_FC = −1.73) and jasmonoyl-valine (JA-Val, log_2_FC = −1.06) were downregulated in MFF.

For gibberellins and ABA, the bioactive gibberellin GA_7_ (log_2_FC = −0.69) and abscisic acid glucose ester (ABA-GE, log_2_FC = −0.80) both showed lower accumulation in MFF.

These findings suggest that altered IAA, CTK, JA, and GA contents may contribute to multi-pistil formation in Chinese chestnut.

### 3.4. Integrated Analysis of Hormonal Targeted Metabolomics and Transcriptomics

**Figure 6:**
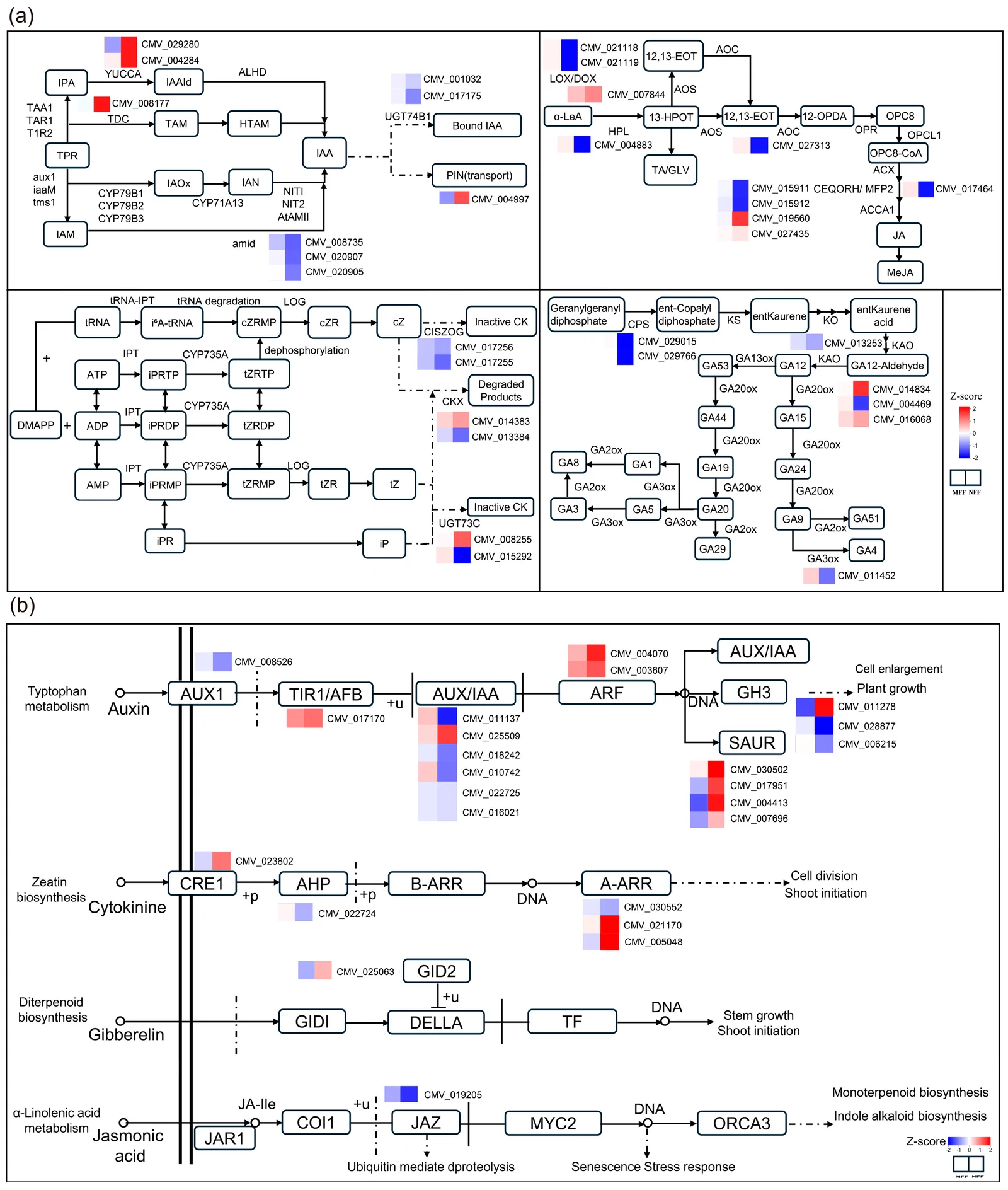
Gene expression profiles of phytohormone biosynthesis, metabolism and signal transduction pathways. (**a**) Gene expression profiles of IAA, CTK, JA and GA biosynthesis and metabolic pathways. (**b**) Gene expression profiles of IAA, CTK, JA and GA signaling pathways. Solid arrows represent enzymatic reaction, substance conversion or positive regulation; dashed arrows indicate transport, indirect or minor metabolic reactions; circles represent transcription factor binding sites on DNA; t-bar lines indicate protein inhibition or degradation; symbols +u and +p denote ubiquitination and phosphorylation modification, respectively. Heatmap squares show gene expression based on Z-score: red for up-regulation, blue for down-regulation.

To explore the association mechanisms between DEGs and DAMs during the differentiation of MFF and NFF, we integrated transcriptomic and metabolomic datasets, focusing on phytohormone biosynthesis, metabolism, and signaling pathways (Figure 6).

#### 3.4.1. Key Genes Associated with the Synthesis and Metabolism Pathways of IAA, CTK, JA, and GA

To screen for candidate genes associated with the synthesis of key hormones, the biosynthesis and metabolism pathways of IAA, CTK, JA, and GA were analyzed (Figure 6a). The transcripts per million (TPM) values of each DEG are listed in Table S4.

In the IAA pathway, nine DEGs were identified. In MFF, the IAA synthesis genes *YUCCA* and *TDC*, as well as the polar transport carrier *PIN*, were significantly downregulated. Meanwhile, *amid*, responsible for converting IAM to active IAA, and the IAA-inactivating gene *UGT74B1* were concurrently upregulated.

In the CTK pathway, six DEGs were identified. Two *CISZOG* genes were significantly upregulated in MFF, whereas *UGT73C* and *CKX* showed differential expression, collectively maintaining CTK homeostasis.

In the JA pathway, ten DEGs were identified. In MFF, the key precursor synthesis enzymes *LOX* and *AOS*, as well as the downstream *MFP2*, were upregulated. *HPL*, which channels the precursor 13-HPOT into a competitive metabolic branch, was significantly upregulated, while *DOX* was significantly downregulated. Additionally, four *CEQORH* genes were differentially expressed.

In the GA pathway, seven DEGs were identified. Two *CPS* genes, one *GA3ox* and one *KAO* were significantly upregulated. Among three GA20ox homologs, two were downregulated and one was upregulated.

#### 3.4.2. Key Genes Associated with IAA, CTK, JA, and GA Signaling Pathways

DEGs associated with the IAA, CTK, JA, and GA signaling pathways were analyzed (Figure 6b). The TPM values of each DEG are listed in Table S5.

In the IAA signaling pathway, 17 DEGs were identified. In MFF, the auxin influx carrier AUX1 was significantly upregulated. Meanwhile, one receptor gene *AFB* and two core transcription factors *ARF* were significantly downregulated. Additionally, six core transcriptional repressors *AUX/IAA* were significantly upregulated, four downstream key response factors *SAUR* were significantly downregulated, and *GH3* genes were differentially expressed.

In the CTK signaling pathway, five DEGs were identified. In MFF, the gene encoding cytokinin receptor *CRE1* was downregulated, whereas the gene for histidine phosphotransfer protein *AHP* was upregulated. Two type-A *ARR* genes, *ARR4* (CMV_021170) and *ARR15* (CMV_005048), were significantly downregulated. By contrast, *ARR17* (CMV_030552) was significantly upregulated.

In the JA and GA signaling pathways, one DEG was identified in each. *JAZ* was upregulated, negatively regulating JA signaling intensity, while *GID2* was downregulated, suppressing GA signaling intensity.

#### 3.4.3. Transcription Factor Analysis

**Figure 7:**
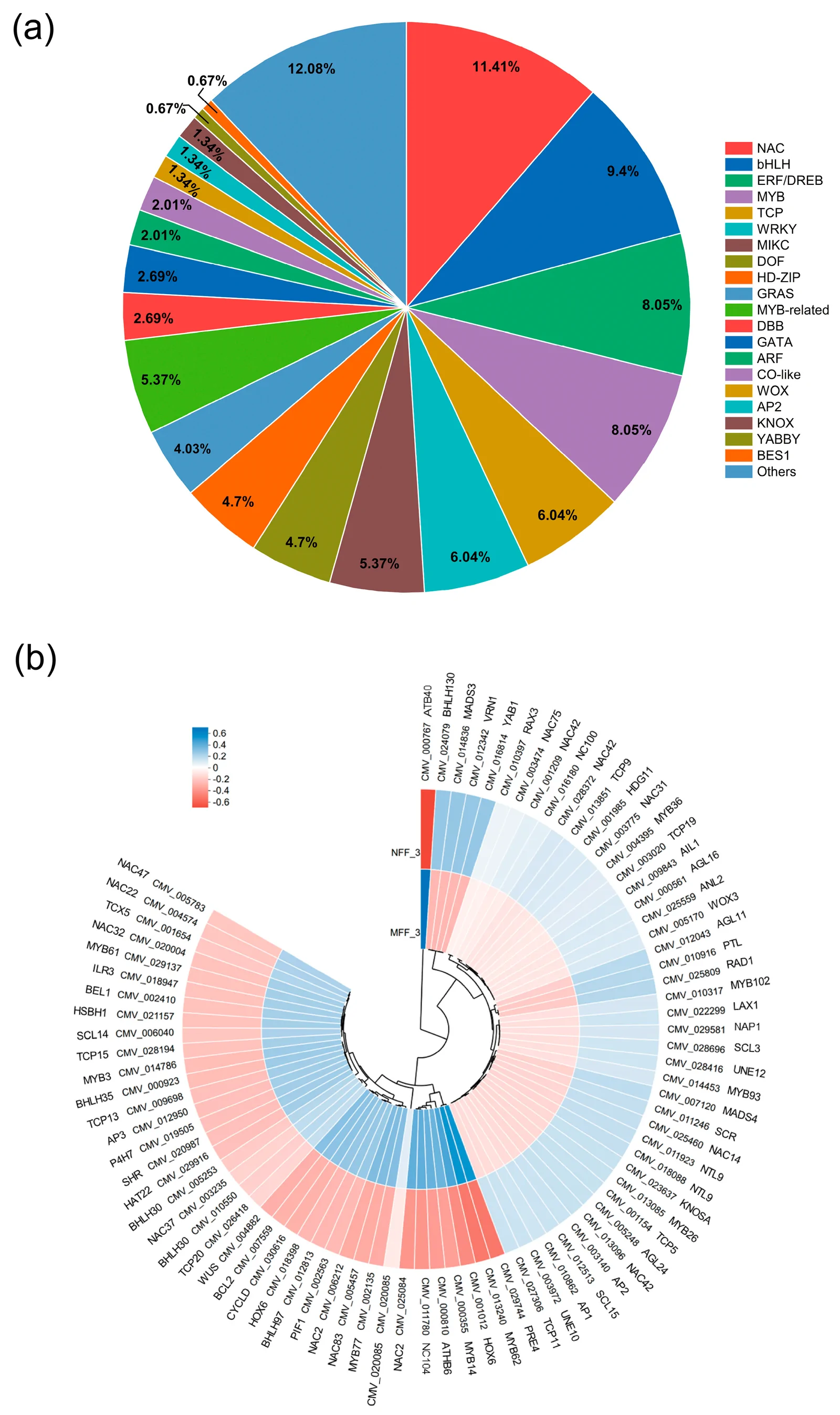
Statistics of differentially expressed TFs. (**a**) Distribution of TFs by family. (**b**) Heatmap of differentially expressed TFs.

In this study, 149 differentially expressed transcription factors (TFs) belonging to 31 families were identified between MFF and NFF (Figure 7a). Among these, the *NAC* family had the largest number of members (17, 11.41%), followed by *bHLH* (14, 9.40%), *ERF/DREB* (12, 8.05%) and *MYB* (12, 8.05%). Additionally, multiple TFs involved in meristem activity, floral organ development, and organ boundary establishment were also identified (Figure 7b, Table S6), implying that these TF families may contribute to multi-pistil formation in Chinese chestnut.

With respect to meristem activity regulation, the *WOX* family gene *WUS* (log_2_FC = +5.50) was significantly upregulated in MFF, while *WOX3* was downregulated. *AP2* and *AIL1* of the *AP2* family were downregulated. In the *KNOX* family, *HSBH1* was upregulated and *KNOSA* was downregulated, suggesting that floral meristem homeostasis may be altered in MFF. Among the *MIKC-type MADS-box* genes, eight were differentially expressed, with seven downregulated and only *AP3* upregulated in MFF. C-class *AG* homologs *AGL11* and *MADS3* (log_2_FC = −4.29 and −2.28, respectively) showed substantial downregulation. These two genes are closely associated with the maintenance of floral meristem determinacy, suggesting that FM termination may be compromised. In addition, the flowering regulator *AGL24* and the floral organ development gene *AP1* were also downregulated. Regarding cell proliferation and organ boundary establishment, differentially expressed members of the *NAC* and *TCP* families were identified. These differentially expressed TFs may regulate the establishment of additional floral meristem boundaries and cell proliferation.

### 3.5. Validation by qRT-PCR

To confirm the gene expression results obtained from RNA-seq data, we selected 14 DEGs for qRT-PCR validation. These DEGs covered the biosynthesis, metabolism, and signaling pathways of IAA, CTK, JA, and GA, as well as transcription factors associated with meristem activity and floral organ development.

**Figure 8:**
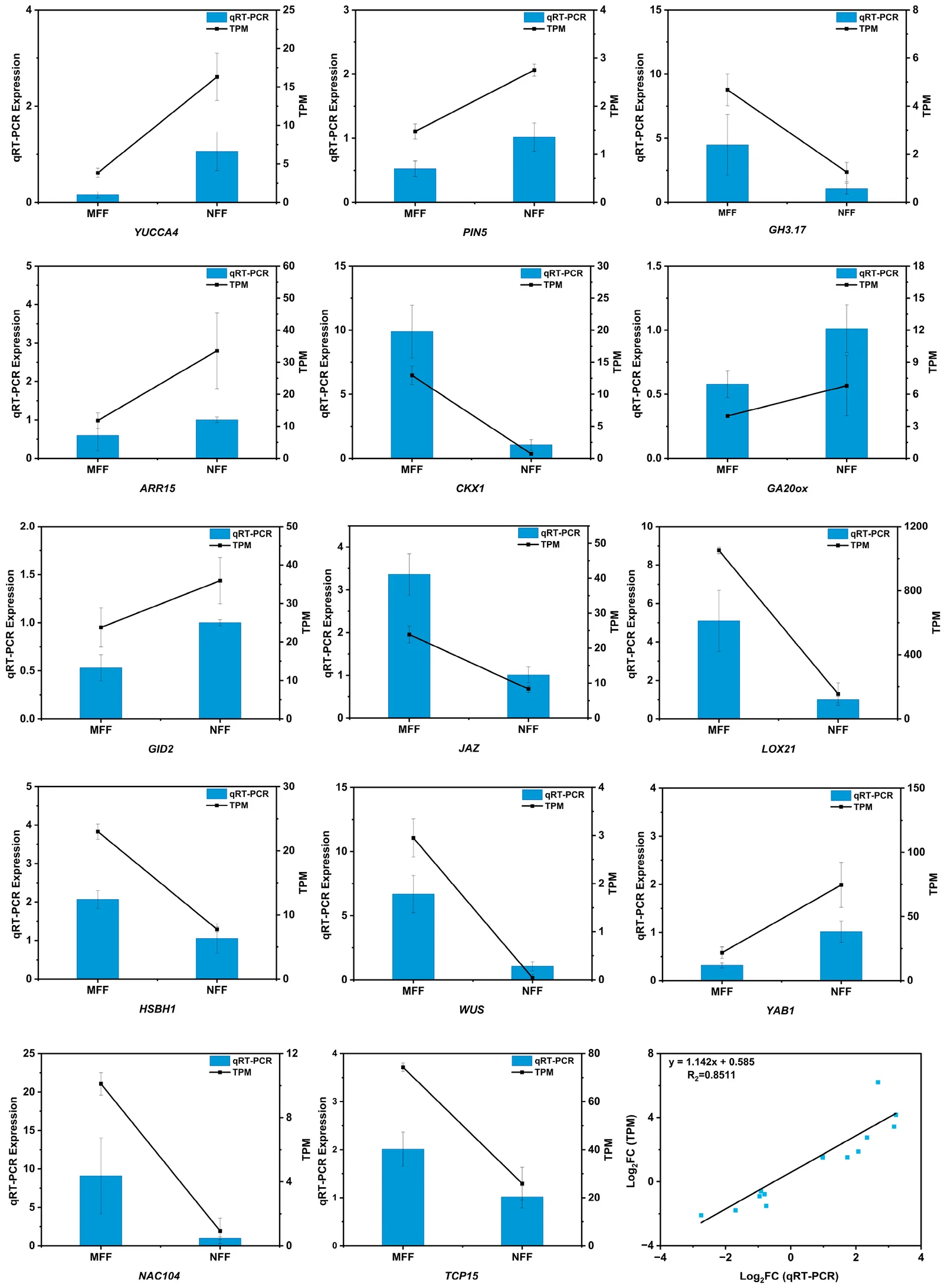
Validation of 14 differentially expressed genes (DEGs) using qRT-PCR. Data are shown as means of three biological replicates. The bar chart indicates TPM values derived from RNA-seq, and the line graph shows relative expression values obtained by qRT-PCR. Error bars denote the standard deviation (SD) of three biological replicates.

In MFF vs. NFF, the qRT-PCR results showed that eight genes (*YUCCA4*, *PIN5*, *CKX1*, *GA20ox*, *GID2*, *YAB1*, *TCP15*, and *NAC104*) were significantly downregulated in MFF, while the other six genes (*GH3.17*, *ARR15*, *LOX1*, *JAZ*, *WUS*, and *HSBH1*) were significantly upregulated in MFF. According to the results of RNA-seq and qRT-PCR, the expression trends of these 14 DEGs were similar, with a linear correlation coefficient of R^2^ = 0.8511 (Figure 8), confirming the reliability and accuracy of the transcriptome analysis.

## 4. Discussion

MFF and NFF showed marked differences in morphology and anatomical structure. Morphological divergence between the two types initiated at the early developmental stage of mixed inflorescences. Specifically, MFF possessed longer basal bracts covered with sparse trichomes that entirely encased the inflorescence, whereas NFF exhibited shorter basal bracts with dense hairs and exposed apical meristems. During subsequent development, the basal bracts of MFF remained significantly longer than those of NFF until bract curling and abscission occurred. These phenotypic characteristics provide a theoretical basis for the early identification of MFF and NFF. Anatomically, the pistil primordium differentiation stage was identified as the critical period for MFF formation. During this stage, the additional floral meristems in MFF continued to enlarge and formed new flower primordia, exhibiting active cell division. Studies have shown that meristem activity determines floral organ number [7,36,37]. Research on rice [9], multi-pistil wheat [8], tomato [11], multi-pistil Japanese apricot [3], and multi-locule rapeseed [5] has demonstrated that meristem proliferation and abnormalities affect floral organ number, leading to multi-pistil or multi-locule phenotypes. Furthermore, enhanced meristem activity and vigorous cell proliferation have been reported in multi-locule rapeseed [38] and multi-pistil wheat [39], which are consistent with the observations obtained in the present study.

Endogenous hormones are key regulators of floral organ development and meristem activity [15]. Previous studies have reported that traits including multi-pistil formation and female flower dominance are frequently correlated with perturbed endogenous hormone homeostasis [3,16,18,19,40]. For instance, multi-pistil inflorescences in coconut accumulate lower IAA, ZR, and GA but higher JA and ABA [16]. Multi-pistil Japanese apricot has elevated ZT and IAA alongside reduced GA_3_ [3]. Branched-flower buds of water lily (*Nymphaea prolifera* Wiersema) contain less CTK, GA_4_ and IAA during bud differentiation [41], while the double-flower buds of *Sagittaria sagittifolia* L. show higher IAA but lower JA and GA relative to the single ones [42]. Collectively, these findings suggest that hormonal changes associated with floral organ and meristem structural variation are highly species-specific. In this work, MFF exhibited decreased IAA precursors (IPA, IAA-Phe) and increased inactivated products (IAA-Gly, oxIAA), whereas bioactive IAA remained unchanged, suggesting altered IAA metabolic patterns. Furthermore, bioactive CTK (iP) and its derivatives, GA_7_, JA-Ile, and JA-Val were significantly decreased and free JA was increased, reflecting perturbed hormone homeostasis in MFF. This hormonal profile shares partial similarities with multi-pistil coconut and branched-flower water lily [16,41]. Combined with previous reports, these coordinated multi-hormone changes may be associated with multi-pistil formation.

IAA and CTK are key regulators of floral meristem activity and floral organ development [43]. IAA governs organ primordium initiation and patterning [44,45], while CTK maintains stem cell activity and meristem proliferation [46,47]. In *Arabidopsis*, elevated IAA attenuates CTK biosynthesis and signaling to terminate floral meristem activity [48,49]. In Japanese apricot, downregulation of *A-ARR* genes and alterations in CTK signaling have been associated with the multi-pistil phenotype [3]. As negative feedback regulators of CTK signaling, *A-ARR* genes promote meristem activity upon their downregulation [50], a phenomenon also observed in MFF, where most *A-ARR* genes were downregulated. In this work, IAA and CTK exhibited divergent changes in MFF. IAA metabolic flux was altered (precursors decreased and inactivation products accumulated), but active IAA levels were unchanged, and signaling appeared suppressed (downregulated *AFB*, *ARF*, and *SAUR*, upregulated *AUX/IAA*). CTK, by contrast, showed reduced content, accompanied by upregulated *CISZOG* and differential expression of *UGT73C* and *CKX*. Its signaling was also altered, with *CRE1* downregulated and *A-ARR* showing overall downregulation. These results suggest a potential perturbation of IAA-CTK homeostasis during pistil primordium differentiation. Similar homeostatic perturbations in IAA and CTK have been observed in other multi-pistil or branched-flower systems [3,16,41], suggesting that altered IAA and CTK homeostasis may be associated with floral meristem activity, although the regulatory mechanisms await further investigation.

Studies have demonstrated that low concentrations of GA can sustain meristem activity and promote cell proliferation [51]. The negative regulatory relationship between KNOX (KNOTTED1-like homeobox) proteins and GA constitutes a conserved pathway for plant meristem homeostasis. KNOX proteins repress *GA20ox* expression to decrease endogenous bioactive GA levels and the undifferentiated state of meristems [52]. This regulatory pattern has been verified in potato (*Solanum tuberosum* L.) [53], tobacco (*Nicotiana tabacum* L.) [54], maize (*Zea mays* L.) [55], and rice [56]. In this study, MFF showed a significant reduction in bioactive GA_7_ content. Although early GA biosynthetic genes including *CPS*, *GA3ox*, and *KAO* were upregulated, the downregulation of *GA20ox* homologs may constrain the accumulation of bioactive GA. Meanwhile, *GID2* was downregulated, implying altered GA signaling output. These molecular and metabolic alterations are consistent with the notion that low GA levels sustain meristem proliferative activity [52]. Furthermore, the KNOX family gene *HSBH1* was significantly upregulated in MFF, displaying an opposite expression trend to *GA20o*x and GA_7_ abundance. These results suggest a potential correlation between the negative regulatory relationship of KNOX and GA and the persistent proliferation of extra floral meristems during pistil primordium differentiation.

In addition, KNOX genes can integrate GA and CTK signals to co-modulate meristem development [57]. High KNOX gene expression coincides with lower GA and higher CTK levels to sustain SAM activity [57]. In *Prunus* species, KNOX genes promoter carry CTK-responsive elements, suggesting a potential bidirectional regulatory relationship between KNOX and CTK [58]. Nevertheless, hormone networks mediated by KNOX genes are not fully conserved, and their regulatory outputs depend on species, meristem types, and developmental contexts [59]. Unlike the canonical antagonistic pattern in *Arabidopsis*, both GA and CTK content levels were reduced in MFF. Comparable findings have been documented in coconut multi-pistillate inflorescences [16] and double-flower buds of *Sagittaria sagittifolia* [42]. These observations suggest that GA and CTK homeostasis during abnormal floral organ formation varies among species and may not strictly conform to the regulatory mode described in *Arabidopsis*. The simultaneous reduction in GA and CTK may be potentially associated with multi-pistil formation in chestnut, which awaits further investigation.

JA and its derivatives have been extensively studied for their functions in floral development [60]. Defects in JA biosynthesis or signaling have been shown to cause floral organ abnormalities across diverse plant species [61,62,63,64,65], and maintaining JA homeostasis is essential for normal floral morphogenesis [20]. In this study, the elevated free JA content in MFF was consistent with the upregulation of biosynthesis genes (*LOX*, *AOS*, *MFP2*). However, the concurrent upregulation of the competing pathway gene *HPL* and the signaling repressor *JAZ* suggested that while JA synthesis was activated, the production of bioactive derivatives (JA-Ile, JA-Val) was compromised, and signal transduction may be suppressed. In rice, JA-deficient mutant *eg1* and *JAZ*-overexpressing lines exhibit impaired floral meristem termination and ectopic floral organ formation [20,66]. Accordingly, upregulation of *JAZ* in MFF suggests a potential correlation with the defective termination of additional floral meristems. Furthermore, JA can antagonize GA signaling by stabilizing DELLA proteins to repress plant growth [67]. Crosstalk also exists between JA and IAA signaling pathways during floral organ development [67]. In J*atropha curcas* L., GA treatment promotes the expression of JA and CTK signaling genes [68]. In MFF, accumulated free JA displayed an opposite trend relative to GA and CTK, while bioactive JA-Ile and JA-Val shared similar trends with GA and CTK. These results suggest potential synergistic or antagonistic interactions between JA and other phytohormones, and such crosstalk may be potentially involved in multi-pistil formation in chestnut.

In this study, several transcription factors were identified as being closely associated with multi-pistil formation. *WUSCHEL* (*WUS*) maintains FM stem cell activity [69], and class I KNOX genes act synergistically with *WUS* to maintain meristem stem cell activity [52]. In this study, the KNOX family gene *HSBH1* and *WUS* were significantly upregulated, suggesting a potential synergistic effect between *HSBH1* and *WUS* in MFF. In Japanese apricot, *PmAGL24* regulates the expression of the KNOX gene *PmKNAT2/6-a* to control pistil number, and overexpression of *PmKNAT2/6-a* leads to multi-pistil formation [14]. Similarly, in this study, *AGL24* was downregulated while *HSBH1* was upregulated in MFF, suggesting a correlated expression pattern among these genes. Additionally, the SHR*-*SCR complex maintains FM stem cell activity [70]. KNOX and BELL genes regulate FM identity and floral primordium boundaries [71]. Differential expression of these genes in MFF may disrupt the boundary definition between floral primordia and FMs, providing conditions for the ectopic initiation of additional floral primordia. *NAC* is a key factor regulating meristem and organ primordium boundary formation [72,73,74]; mutations in *NAC* can lead to floral organ fusion [74]. *TCP20* promotes cell division [75], while *TCP5* suppresses cell division [76]. In MFF, NAC family genes were differentially expressed, with *TCP20* upregulated and *TCP5* downregulated. These changes are potentially correlated with modified cell division profiles and altered organ boundary development. Collectively, these transcription factor expression profiles may be involved in the modulation of multi-pistil formation in Chinese chestnut.

Based on anatomical, targeted phytohormone metabolomic, and transcriptomic data, we constructed a correlation-based hypothetical model to summarize key molecular differences between MFF and NFF specifically at the pistil primordium differentiation stage (Figure S2). This model was established solely based on datasets from a single developmental time point, reflecting only molecular correlations rather than causal or temporal regulatory relationships. The model integrates the major findings of this study: at the hormonal level, MFF exhibited divergent hormone-associated profiles relative to NFF, including altered IAA-CTK homeostasis, species-specific GA-CTK correlation, and imbalanced JA synthesis signaling status. At the combined transcriptional–hormone level, the upregulation of the *HSBH1* coincided with reduced GA levels in MFF. At the transcriptional level, differential expression of key transcription factors was observed, including negatively correlated expression of *AGL24*, which was downregulated, and *HSBH1*, which was upregulated; coordinated upregulation of *HSBH1* and *WUS*; and distinct expression profiles of NAC and TCP family members. Collectively, these coordinated molecular variations in hormone profiles and meristem-related gene expression are potentially correlated with the continuous proliferation and enlargement of additional floral meristems in MFF.

Notably, transcriptomic and phytohormone metabolomic samples in this study were only collected at the pistil primordium differentiation stage, when morphological differences between the two types of female flower clusters had already emerged. Accordingly, our results only reflect divergent hormone and gene expression features at the onset of such morphological divergence. We cannot resolve the temporal sequence of molecular events nor distinguish whether these molecular changes act as upstream triggers for meristem fate specification or represent concomitant responses after phenotypic establishment. Even so, this work aims to identify candidate genes and hormone signals associated with multi-pistil formation at this critical phenotype-determining stage, providing targets for subsequent screening. Furthermore, our hypothetical model is built solely on omics-based correlation analysis without any gene-functional validation. Future work should include dynamic sampling across multiple developmental time points to construct temporal molecular networks, combined with genetic-functional assays, to clarify causal mechanisms within core pathways and refine the molecular framework underlying multi-pistil development in Chinese chestnut.

## 5. Conclusions

This study integrated morphological, anatomical, targeted phytohormone metabolomic, and transcriptomic analyses to identify phytohormone signals and candidate genes associated with the multi-pistil phenotype during the critical pistil primordium differentiation stage and to characterize the physiological and molecular differences between MFF and NFF. Morphological and anatomical observations showed that MFF had longer basal bracts with fewer basal trichomes. Pistil primordium differentiation was identified as the critical period for multi-pistil formation, during which the additional FMs in MFF continuously enlarged and differentiated into new flower primordia. At this stage, MFF showed comprehensive changes in the accumulation and signaling of IAA, CTK, GA, and JA. The synchronized alterations in these four phytohormones and the expression of their related genes reflect perturbed hormone homeostasis in MFF, which is potentially correlated with the multi-pistil phenotype. Furthermore, several transcription factor families, including WOX, KNOX, AP2, NAC, and TCP, exhibited differential expression profiles that may be associated with meristem activity and organ boundary establishment. This work preliminarily characterizes the anatomical differences and the potential associations between phytohormones, genes, and the phenotype at this critical developmental stage, providing a basis for future functional validation. This work offers a theoretical foundation for understanding the potential regulatory mechanisms underlying multi-pistil formation in Chinese chestnut.

## Figures and Tables

**Table 1:** Morphological characteristics of MFF and NFF at different developmental stages.

Date	Morphological Stage	Anatomical Stage	Cluster Type	Basal Bract Length (cm)	Inflorescence Length (cm)
Apr 28	Bract differentiation	Floral cluster primordium differentiation	MFF	0.23 ± 0.03 **	0.24 ± 0.03 *
			NFF	0.18 ± 0.01	0.22 ± 0.02
May 3	Bract enlargement	Flower primordium differentiation	MFF	0.36 ± 0.05 **	0.42 ± 0.04 ns
			NFF	0.26 ± 0.04	0.43 ± 0.04
May 8	Bract elongation	Sepal primordium differentiation	MFF	0.56 ± 0.04 **	0.78 ± 0.10 *
			NFF	0.26 ± 0.02	0.76 ± 0.10
May 13	Inflorescence elongation	Stamen primordium differentiation	MFF	0.65 ± 0.05 **	1.14 ± 0.09 ns
			NFF	0.37 ± 0.03	1.11 ± 0.07
May 16	Female flower formation	Pistil primordium differentiation	MFF	0.73 ± 0.03 **	1.63 ± 0.15 ns
			NFF	0.42 ± 0.06	1.68 ± 0.13
May 19	Female flower swelling	Stigma primordium differentiation	MFF	0.72 ± 0.04 **	4.45 ± 0.59 ns
			NFF	0.44 ± 0.04	4.53 ± 0.64
May 31	Multiple female flower emergence	Ovary formation	MFF	—	9.87 ± 0.79 ns
			NFF	—	10.21 ± 0.81
Jun 7	Stigma bifurcation	Flowering	MFF	—	9.99 ± 0.66 **
			NFF	—	11.57 ± 0.50

Data are means ± SD. Asterisks indicate significant differences between MFF and NFF at the same developmental stage (* *p* < 0.05, ** *p* < 0.01); ns, no significant difference; “—“ means basal bracts abscised and measurements were unavailable. All samples were collected in 2025 on the dates listed.

## Data Availability

All data are open and available. The raw data are available in the NCBI (BioProject ID PRJNA1498628) (https://www.ncbi.nlm.nih.gov/sra/, accessed on 21 July 2026). The data will be made publicly available upon publication of this article.

## Supplementary Materials

The following supporting information can be downloaded at: https://www.mdpi.com/article/10.3390/biology15171459/s1. Table S1: Primers used in qRT-PCR in this work; Table S2: Data filtering and comparison of reference tables; Table S3: Statistics of differentially accumulated phytohormones between MFF and NFF; Table S4: TPM values of DEGs involved in biosynthesis and metabolism pathways of IAA, CTK, JA and GA; Table S5: TPM values of DEGs in signal transduction pathways of IAA, CTK and JA; Table S6: Transcription factors related to meristem maintenance, floral organ development and hormone signaling; Figure S1: Transcriptome overview of female flower clusters in Chinese chestnut. Figure S2: Correlation-based hypothetical model summarizing key hormone and molecular differences between MFF and NFF of Chinese chestnut at the pistil primordium differentiation stage.
