# Supplementary material for: Insights into Candidate Pathways Associated with Multi-Pistil Formation in Chinese Chestnut (Castanea mollissima Bl.): Evidence from Anatomy, Physiology, and Molecular Biology
Source: Biology (Basel). 2026 Aug 26;15(17):1459. doi: 10.3390/biology15171459 (PMC13565177; doi:10.3390/biology15171459)
Supplement: Supplementary file 1 [file biology-15-01459-s001.zip › biology-4487650-supplementary.pdf]

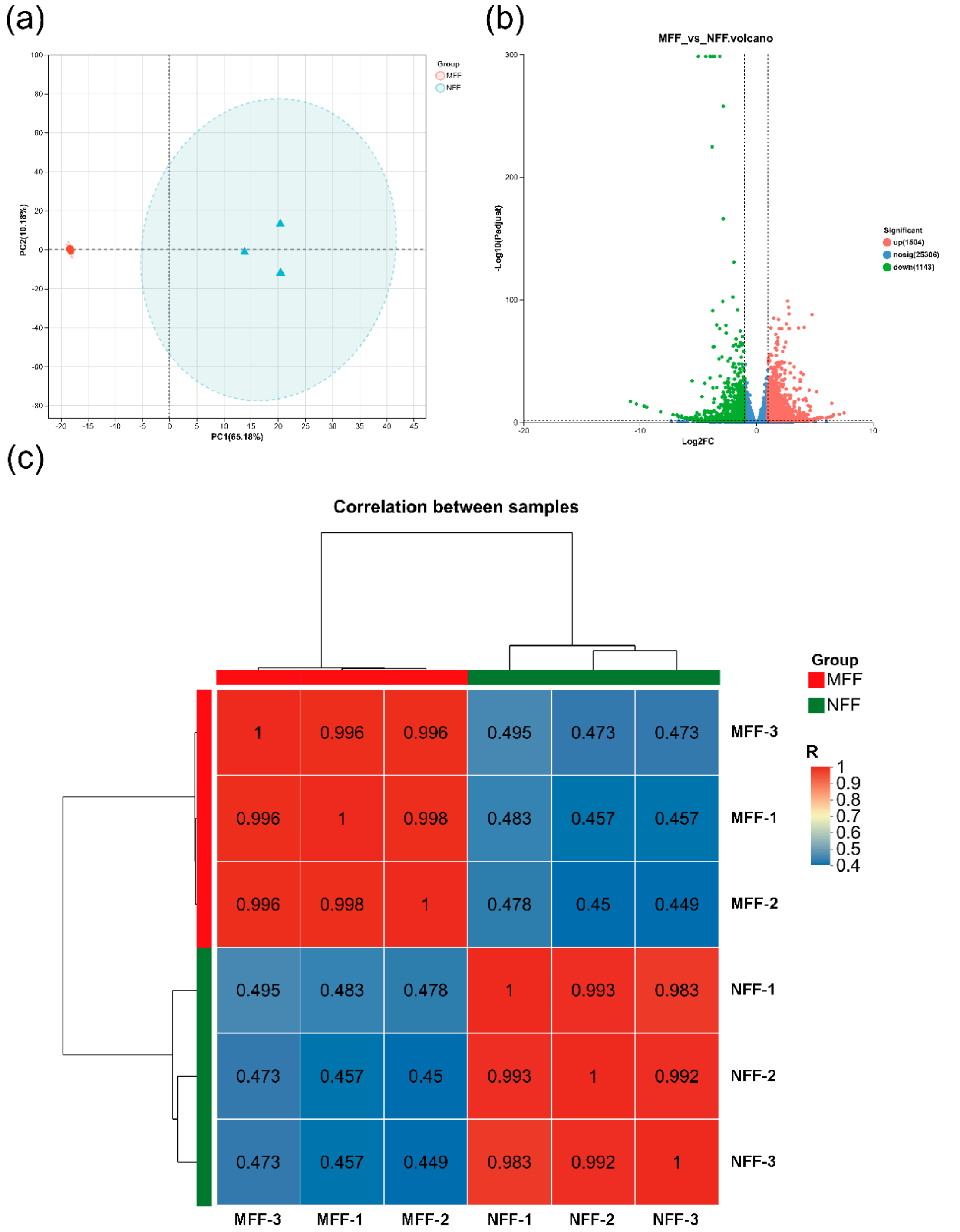

Figure S1. Transcriptome overview of female flower clusters in Chinese chestnut. (a) PCA of transcriptome profiles of MFF and NFF. PC1 explained 65.18% of the total variance, and PC2 explained 10.18%. (b) Statistics of DEGs in MFF vs. NFF. (c) Pearson's correlation coefficient (PCC) heatmap showing pairwise correlations among all transcriptome samples.

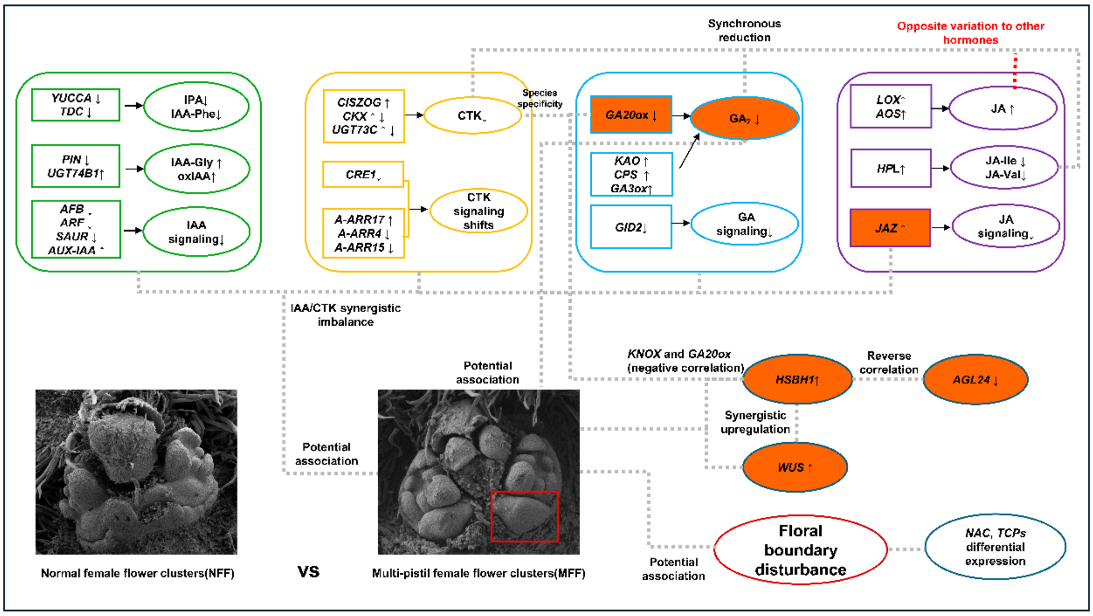

Figure S2. Correlation-based hypothetical model summarizing key hormone and molecular differences between MFF and NFF of Chinese chestnut at the pistil primordium differentiation stage. Up and down arrows indicate upregulation and downregulation of genes or changes in phytohormone abundance in MFF relative to NFF. Solid right-pointing arrows denote observed directional shifts within hormone-related pathways. Grey dashed lines represent potential molecular correlations detected at this sampled developmental time point rather than proven causal regulatory relationships. This schematic integrates anatomical, targeted phytohormone metabolomic and transcriptomic data and illustrates candidate hormone and molecular features associated with the multi-pistil phenotype.
